# Updates to the Melbourne Children’s Regional Infant Brain Software Package (M-CRIB-S)

**DOI:** 10.1007/s12021-024-09656-8

**Published:** 2024-03-16

**Authors:** Chris L. Adamson, Bonnie Alexander, Claire E. Kelly, Gareth Ball, Richard Beare, Jeanie L. Y. Cheong, Alicia J. Spittle, Lex W. Doyle, Peter J. Anderson, Marc L. Seal, Deanne K. Thompson

**Affiliations:** 1 Royal Children’s Hospital, Murdoch Children’s Research Institute, Flemington Road, Parkville, Victoria 3052 Australia; 2 Turner Institute for Brain and Mental Health, School of Psychological Sciences, Monash University, Melbourne, Australia; 3 Department of Medicine, Monash University, Melbourne, Australia; 4 Neonatal Services, The Royal Women’s Hospital, Melbourne, Australia; 5 Department of Obstetrics and Gynaecology, The University of Melbourne, Melbourne, Australia; 6 Department of Physiotherapy, The University of Melbourne, Melbourne, Australia; 7 Department of Paediatrics, The University of Melbourne, Melbourne, Australia; 8 Florey Department of Neuroscience and Mental Health, The University of Melbourne, Melbourne, Australia

**Keywords:** Neonate, Baby, Segmentation, Magnetic resonance imaging, Cortical, Sulcus, Gyrus

## Abstract

The delineation of cortical areas on magnetic resonance images (MRI) is important for understanding the complexities of the developing human brain. The previous version of the Melbourne Children's Regional Infant Brain (M-CRIB-S) (Adamson et al. *Scientific Reports, 10*(1), 10, 2020) is a software package that performs whole-brain segmentation, cortical surface extraction and parcellation of the neonatal brain. Available cortical parcellation schemes in the M-CRIB-S are the adult-compatible 34- and 31-region per hemisphere Desikan-Killiany (DK) and Desikan-Killiany-Tourville (DKT), respectively. We present a major update to the software package which achieves two aims: 1) to make the voxel-based segmentation outputs derived from the *Freesurfer*-compatible M-CRIB scheme, and 2) to improve the accuracy of whole-brain segmentation and cortical surface extraction. Cortical surface extraction has been improved with additional steps to improve penetration of the inner surface into thin gyri. The improved cortical surface extraction is shown to increase the robustness of measures such as surface area, cortical thickness, and cortical volume.

**Supplementary Information:**

The online version contains supplementary material available at 10.1007/s12021-024-09656-8.

## Introduction

Understanding human brain development requires accurate delineation of anatomical structures and parcellation of the cerebral cortex from around the time of birth. Computational methods applied to brain magnetic resonance images (MRI) can be used for this purpose. A study may utilize a cross-sectional or longitudinal design at multiple ages to assess developmental trajectories of brain structure geometric properties.

*FreeSurfer* (Fischl, 2012; Fischl & Dale, 2000; Fischl et al., 2002, 2004) is a commonly used whole-brain anatomical labeling, cortical extraction and parcellation software package applicable to *T*_1_-weighted MRI scans of children and adults. *FreeSurfer*’s available cortical parcellation schemes include the Desikan-Killiany (DK) (Desikan et al., 2006) and Desikan-Killiany-Tourville (DKT) (Klein & Tourville, 2012) adult atlases. Applying *FreeSurfer* to brain images with adult-like tissue contrast within a cross-sectional or longitudinal study yields accurate results. However, tools tuned for adult brains, such as the adult *T*_1_-based templates and atlases provided in *FreeSurfer* (Fischl et al., 1999, 2004), are not directly applicable to neonatal brain images. There are inherent differences in anatomy and tissue composition between infant and adult brains. Primarily, tissue signal intensities are different in neonatal brains compared with those in adults (Wang et al., 2019). Thus, the optimal MRI sequences for the gray and white matter contrast required to extract cortical surface boundaries differ based on age. While *T*_*1-*_weighted contrasts are optimal for adult brains, *T*_*2*_-weighted contrasts are optimal for neonatal brains. Consequently, specialized algorithms are required to contend with neonatal-specific tissue intensities (Beare et al., 2016; Makropoulos et al., 2016). Thus, brain atlases and image segmentation and parcellation tools specific for infants are required. We have produced infant-specific atlases, M-CRIB (Alexander et al., 2017) and M-CRIB 2.0 (Alexander et al., 2019), to match the DK and DKT parcellation schemes, respectively.

**Fig. 1:**
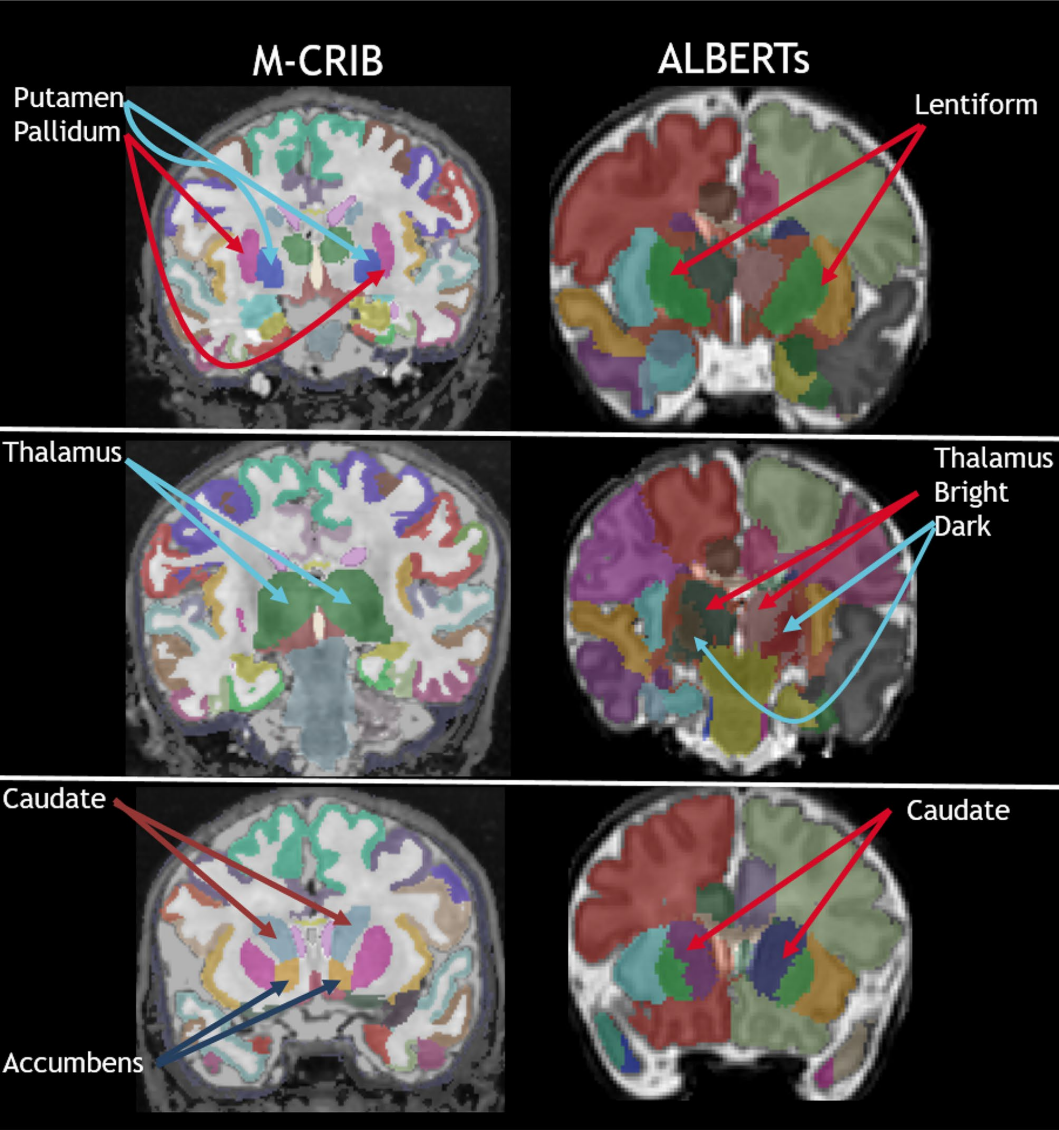
Depiction of some voxel-based segmentation labels for the Freesurfer-compatible M-CRIB (used in this software version) and the Freesurfer-incompatible ALBERTs (used in the previous software version) schemes that are incompatible

Based on the M-CRIB (Alexander et al., 2017) and M-CRIB 2.0 (Alexander et al., 2019) atlases, we released the *M-CRIB-S* software (Adamson et al., 2020), which performed the following sequence of steps: 1) whole-brain labelling using *DrawEM* (Makropoulos et al., 2014), 2) cortical surface extraction using *Deformable* (Schuh et al., 2017), and 3) cortical parcellation into DK and DKT schemes using a *Freesurfer*-like pipeline. This previous software version had two limitations which are addressed here. First, the whole-brain segmentations were produced using the *Freesurfer*-incompatible *ALBERTs* (Gousias et al., 2012; Makropoulos et al., 2014) scheme. This meant that non-cortical regions labeled with the M-CRIB-S at the neonatal time point, could not be compared with non-cortical regions labeled using *FreeSurfer* at older time points; for some examples see Fig. 1. Second, during cortical surface extraction, the grey-white boundary often failed to penetrate thin white matter strands. White matter was misclassified as grey matter in such regions, leading to errors in surface geometry. This paper introduces a major update to the software to address these issues with the following changes:

1. An M-CRIB-based whole-brain labelling that is compatible with adult Freesurfer, with components as follows:
  An M-CRIB template space containing *T*_*2*_-, *T*_*1*_-weighted and label images.
  A label fusion method that utilizes *ANTs* (Tustison et al., 2014) for registration, *DrawEM* for initialization, and *antsJointFusion* (Wang & Yushkevich, 2013) for final labelling.
2. An improved cortical surface extraction technique that includes force terms to encourage penetration of the surface into thin gyri.

## Methods

**Fig. 2:**
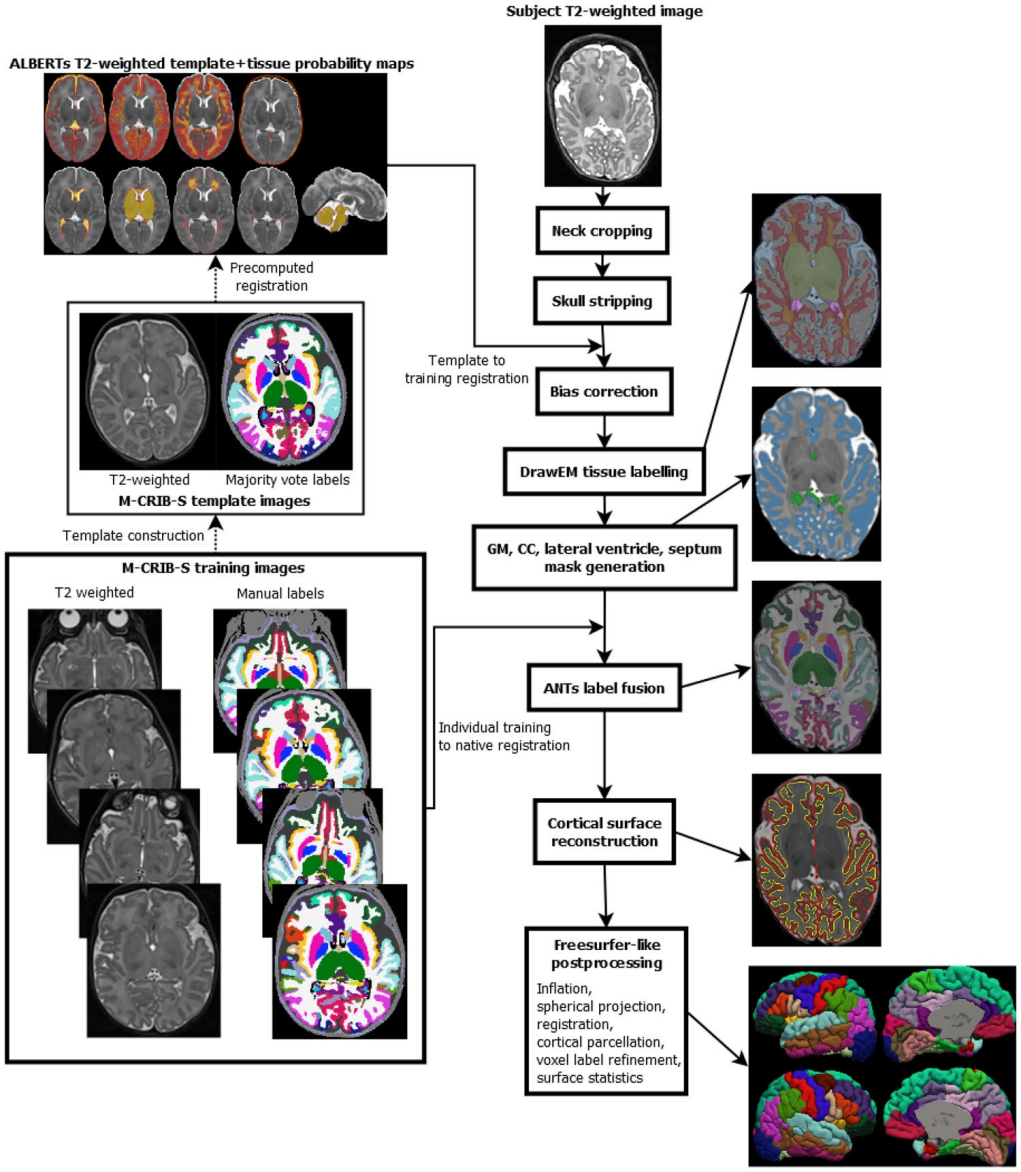
Depiction of the pipeline employed by the new version of the software. After neck cropping, skull stripping, and bias correction, the ALBERTs 40-week template and posterior fissure mask are registered to subject native space followed by DrawEM tissue labelling into 9 classes. The DrawEM tissue labels and majority vote M-CRIB labels are used to form approximate GM, CC, lateral ventricle masks. All 10 M-CRIB training intensity and label images are registered to the native image which are used to perform ANTs label fusion. Cortical surface reconstruction, using a Deformable-based method is performed. Finally, a Freesurfer-like pipeline incorporating cortical surface inflation, spherical projection, registration, parcellation with vertex- and DKT-region-wise thickness, surface area, volume measures is performed

Figure 2 depicts the overall M-CRIB-S pipeline, including voxel labelling, cortical surface extraction, refined voxel labelling and cortical surface parcellation. The voxel labeling step segments per-hemisphere cortical gray and white matter (GM/WM); subcortical gray nuclei; cerebellar, brainstem, and ventricular structures; and cerebrospinal fluid (CSF). Cortical surface extraction estimates the inner (“white”) and outer (“pial”) cortical surface boundaries using triangular meshes. Cortical parcellation is performed using a Freesurfer-like pipeline that involves: 1) surface inflation and spherical projection, 2) surface-based registration to a spherical template, and 3) per-vertex labelling based on template-space training data. A description of the training data will be given below, then a description of the method will follow.

### Training Data

The term ‘training data’ denotes the collection of images used for any voxel labelling, image-based standard spaces, and surface templates. Training data were taken from two main collections: ALBERTs and M-CRIB-S. Here, the term “ALBERTs” refers to the 40-week image template and its corresponding 9-tissue type probability maps, described previously (Gousias et al., 2012; Makropoulos et al., 2014). The *ALBERTs* were used for the voxel-based labelling in the previous iteration of the software (Adamson et al., 2020). The M-CRIB-S training data have also been described previously (Adamson et al., 2020; Alexander et al., 2017, 2019). The voxel-based M-CRIB and M-CRIB 2.0 labels include cerebellar hemispheres and vermis, *Freesurfer*-compatible subcortical gray nuclei (Loh et al., 2016), and Desikan-Killiany and Desikan-Killiany-Tourville compatible parcels of the cerebral cortex (Alexander et al., 2017, 2019). A major component of this paper involves modifications to the M-CRIB training data to improve labeling and parcellation results; these changes will be described below.

A total of 53 term-born (≥ 37 weeks’ gestation), healthy neonates (40.2 – 44.9 weeks’ postmenstrual age (PMA) at scan, M = 42.3, SD = 1.2, 24 female) were scanned as control subjects for studies examining the impact of preterm birth on brain development (Spittle et al., 2014; Walsh et al., 2014). Criteria for control selection were: 1) no admissions to a neonatal intensive care or special care unit, 2) did not receive resuscitation at birth, 3) birthweight more than 2.5 kg, and 4) no evidence of congenital conditions known to affect development and growth. Written, informed consent was obtained from parents. Ethical approval for the studies was obtained from the Human Research Ethics Committees of the Royal Women’s Hospital and the Royal Children’s Hospital, Melbourne and the research studies complied with the standards of the Declaration of Helsinki.

All subjects were scanned at the Royal Children’s Hospital, Melbourne, Australia, on a 3T Siemens Magnetom Trio scanner during unsedated sleep. *T*_*2*_-weighted images were acquired with a turbo spin echo sequence with the following parameters: 1 mm axial slices, flip angle = 120°, repetition time = 8910 ms, echo time = 152 ms, field of view = 192 × 192 mm, in-plane resolution = 1 mm^2^ (zero-filled interpolated to 0.5 × 0.5 × 1 mm in image reconstruction), matrix size = 384 × 384. *T*_*1*_-weighted images were acquired with a spoiled, gradient-recalled, inversion-recovery sequence with the following parameters: 1 mm axial slices, flip angle = 9°, repetition time = 2100 ms, inversion time = 1100 ms, echo time = 3.39 ms, field of view = 174 × 192 mm, matrix size = 348 × 384, in-plane resolution = 1 mm^2^ (zero-filled interpolated to 0.5 × 0.5 × 1 mm in image reconstruction). All *T*_*2*_- and *T*_*1*_-weighted images were resliced to the isotropic voxel-volume-preserving size of 0.63 mm^3^ (Alexander et al., 2017; Loh et al., 2016).

This cohort was subdivided into the following two subsets: *labelled* and *unlabelled* subsets. The *labelled* set comprised the ten M-CRIB subjects (Alexander et al., 2017, 2019) (40.29 – 43.00 weeks’ PMA at scan, M = 41.71, SD = 1.31; 4 female). The *unlabelled* subset consisted of the remaining 43 subjects (40.3 – 44.9 weeks’ PMA at scan, M = 42.4, SD = 1.2, 20 female).

Some manual edits to the existing M-CRIB 2.0 volumetric training data were performed the author (C.A.), including definition of additional labels, for the purpose of improving segmentation outputs. These were as follows:

1. For two of the 10 training data subjects, T2 images had initially been cropped such that the full extent of the superior aspect of the brain was not captured, and some gyral crowns and CSF were omitted. Uncropped images for these subjects were retrieved, and previously cropped slices had relevant labels manually painted in.
2. Two non-brain labels: an “Extra-cranial background” label and a bright non-brain label “skull”, were added to assist the label fusion algorithm in segmenting non-brain voxels that were not removed by the skull stripping step. The “Extra-cranial background” label comprised all voxels within 3 mm of the brain mask boundaries. The “skull” label covered the bright voxels outside the brain, according to the negative values of a 2nd derivative filter applied outside the brain mask.
3. New labels were introduced to aid processing steps and to produce segmentations with increased detail. These labels were:
  - The dark periventricular stripes surrounding the lateral ventricles; these labels were not included in the final outputs. Anatomically, this label approximately represents the tapetum and it extends to the lateral and superior extrema of the lateral ventricles.
  - The left/right choroid plexus and left/right septum pellucidum (SP) were manually drawn on training data. These labels were included in final outputs. However, the reliability and accuracy of these labels have not been evaluated. In order to obtain lateral ventricle volumes from segmentation outputs, it is necessary to add the choroid plexus and lateral ventricle label volumes.

Cortical parcellation training data was reconstructed by incorporating the revised data in the same fashion as the previous work (Adamson et al., 2020).

### Template Construction

The M-CRIB-S template is a common-space averaged version of all M-CRIB training subjects. The M-CRIB-S template space was built using the ANTs script antsMultivariateTemplateConstruction2.sh, which performs iterative registration of native space data to the previous iteration’s template space followed by averaging to create a new template space. The initial template space was created by aligning 9 of the 10 subjects *T*_*2*_-weighted images to that of one subject, that had a high-quality image per visual inspection, using rigid + affine registration with Mutual Information (MI) cost function. 

**Fig. 3:**
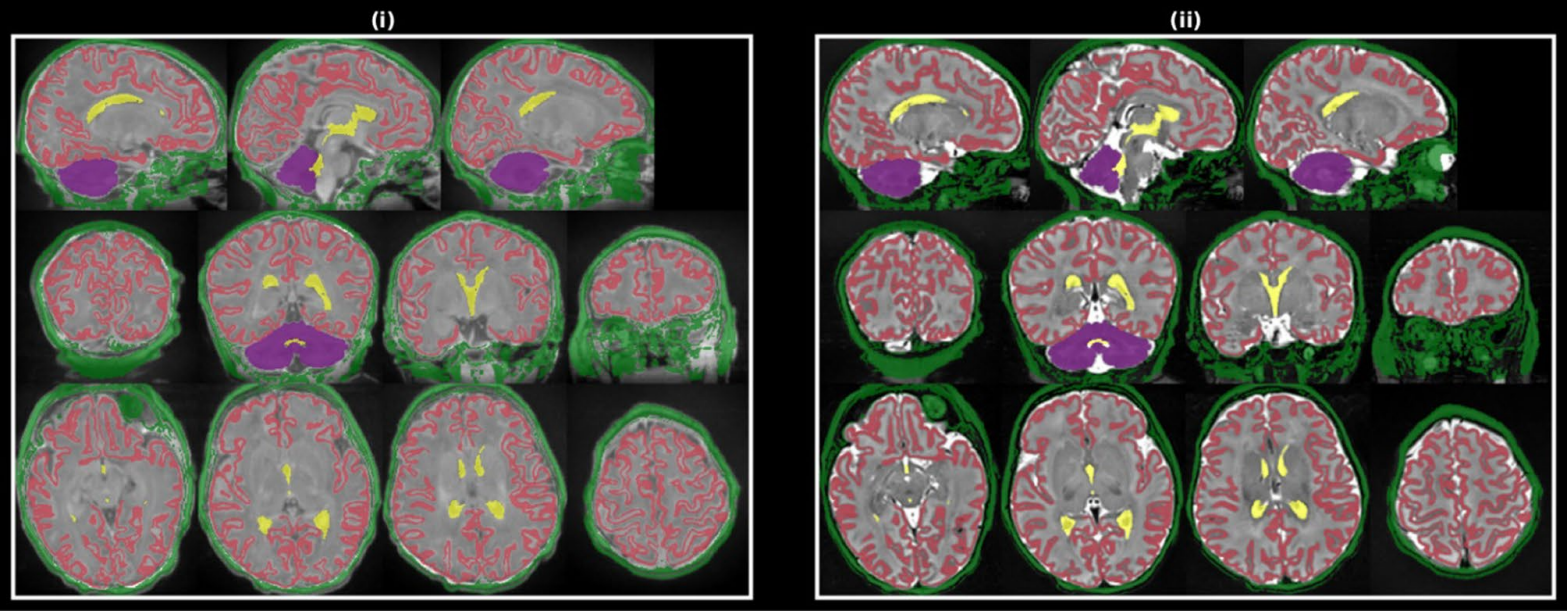
One training subject’s T_1_-weighted (i) and T_2_-weighted (ii) images with structure label masks used for template construction registration colored as follows: cerebral GM (red), Cerebellum (purple), “Skull” (green), Lateral Ventricles and central CSF (yellow)

Registration of native space data to the previous iteration template space was performed with ANTS using a rigid + affine + SyN (Avants et al., 2008) transformation sequence. The image registration pairs consisted of intensity and label images, where label images were used to maximize alignment of anatomical landmarks, particularly for structures with high degrees of inter-subject variability of shape and location. Specifically, the image channels were *T*_*2*_- and *T*_*1*_-weighted images, and the label channels were lateral ventricles, cerebellum, cortical GM, “scalp” or outer bright (see Fig. 3). Registration cost functions were MI for intensity image pairs and Thirion’s Demons (Thirion, 1998) for label images. A template-space brain mask probability map and majority vote label image were derived from all 10 *T*_*2*_-weighted and label images after transformation into template space.

An ANTs affine and non-linear warp (SyN) from the 40-week *ALBERTs T*_*2*_-weighted template image to the M-CRIB-S *T*_*2*_-weighted template image was computed. Here, equally weighted MI and local Cross Correlation (LCC) (2 mm radius) were used as cost functions for the non-linear stage; see Table 1 for a complete list of parameters.

**Table 1:** Parameters for ALBERTs template to M-CRIB-S template ANTs registration routine

Transform	Update field smoothing	Total field smoothing	Iterations	Shrink factors	Smoothing	Cost functions, channels, weighting
Rigid			1000 × 500 × 250 × 100	8 × 4 × 2 × 1	4 × 3 × 2 × 1vox	MI, *T*_*2*_
Affine			1000 × 500 × 250 × 100	8 × 4 × 2 × 1	4 × 3 × 2 × 1vox	MI, *T*_*2*_
SyN	1.5	0	200 × 200 × 50	4 × 2 × 1	2 × 1 × 0vox	MI, *T*_*2*_, 0.5
						LCC, *T*_*2*_, 0.5

### Pipeline for a Novel Image

The full pipeline that results in voxel labeling and cortical surface extraction is shown in Fig. 2.

The process starts by placing a subject, named < foo > , *T*_*2*_-weighted image in the directory RawT2: RawT2/ < foo > .nii.gz. 

The driver script MCRIBReconAll is used to execute a sequence of processing directives that can be executed in isolation or in succession. All MCRIBReconAll calls must run from the same base directory and its synopsis is:

*MCRIBReconAll [processing directives and options] subject id*

For the subject < foo > all calls will be:

*MCRIBReconAll [processing directives and options]* <*foo*>

#### Preprocessing

Neck cropping, skull stripping and bias correction are necessary preprocessing steps, and have processing directives --neckcrop, --skullstrip, --biascorrect respectively.

The neck cropping step firstly reorients the input image to comprise axial slices in radiological format; then aims to find the cropping box that removes slices inferior to the cerebellum, and slices containing only background. The template-space brain mask is then projected onto the native image via linear registration of the *T*_*2*_-weighted image to the M-CRIB-S template. The bounding box formed by the brain mask is then applied to crop the image. Finally, this cropped image is resampled to isotropic voxels of size that is either volume-preserving or user-specified.

**Fig. 4:**
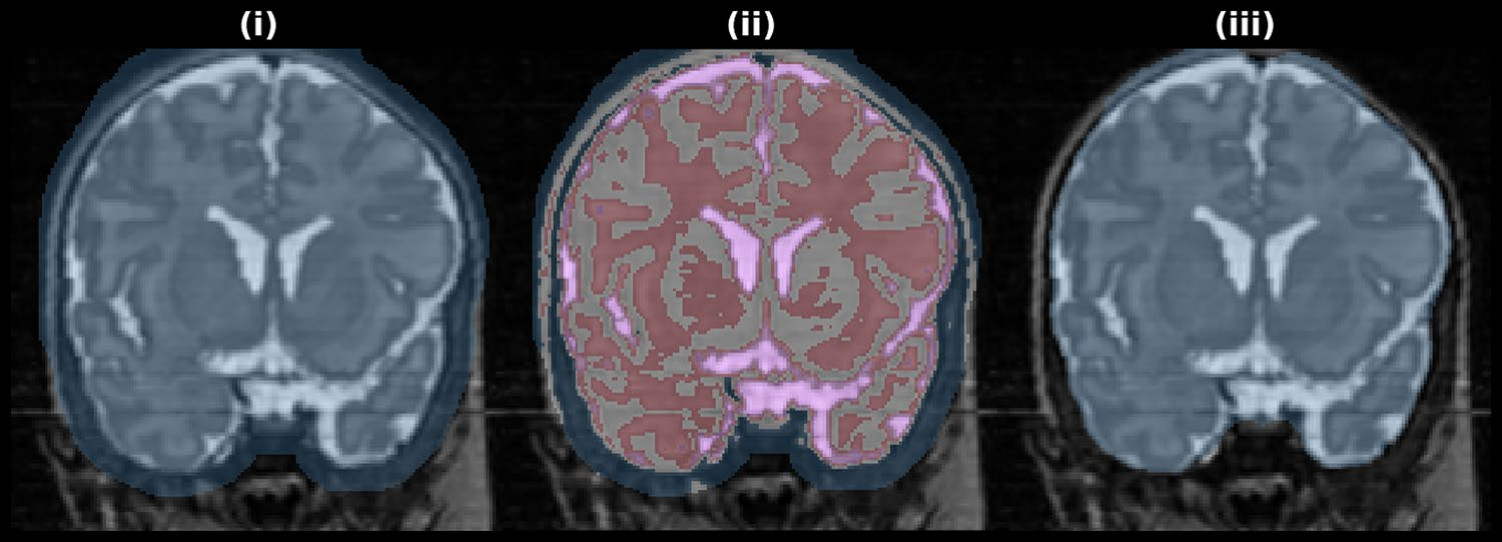
Example of the proposed skull stripping procedure. After BET (i) a 4-class K-means clustering is applied to the intensities within the mask (ii). The outer stripe of the scalp and other non-brain tissue is then mostly removed with a morpholgical opening, largest component, morphological closing to produce the final brain mask (iii)

The skull stripping method comprises the following sequence of steps:

1. The Brain Extraction Tool (BET) (Smith, 2002) is run to generate a brain mask, with a conservative threshold of 0.2 to reduce the chance of brain tissue being omitted by the mask (Fig. 4(i)).
2. The non-brain background tissue remaining in the mask (e.g., scalp, eyes) is minimized using the following steps:
3. A 4-threshold K-Means clustering of the T2 image is applied within the BET mask (Fig. 4(ii)); the darkest class is background, which is removed.
4. On the resultant mask, a binary morphological opening is performed (4 mm spherical structuring element) to disconnect/remove outer bright stripes. The largest connected component is retained, to remove disconnected remaining regions of bright voxels. A binary morphological closing is performed (7 mm spherical structuring element) to smooth the mask boundary (Fig. 4(iii)). The structuring element used for the closing is larger than that used for the opening, to close holes.

Bias correction using *N4* (Tustison et al., 2010) is executed on the whole image whilst restricting the bias field estimation region to the estimated brain mask.

#### Voxel Labelling

The voxel labelling step, processing directive --tissueseg, composed of “Mask generation”, and “Label fusion” in Fig. 2, will now be described.

#### Initial Template Registration

**Fig. 5:**
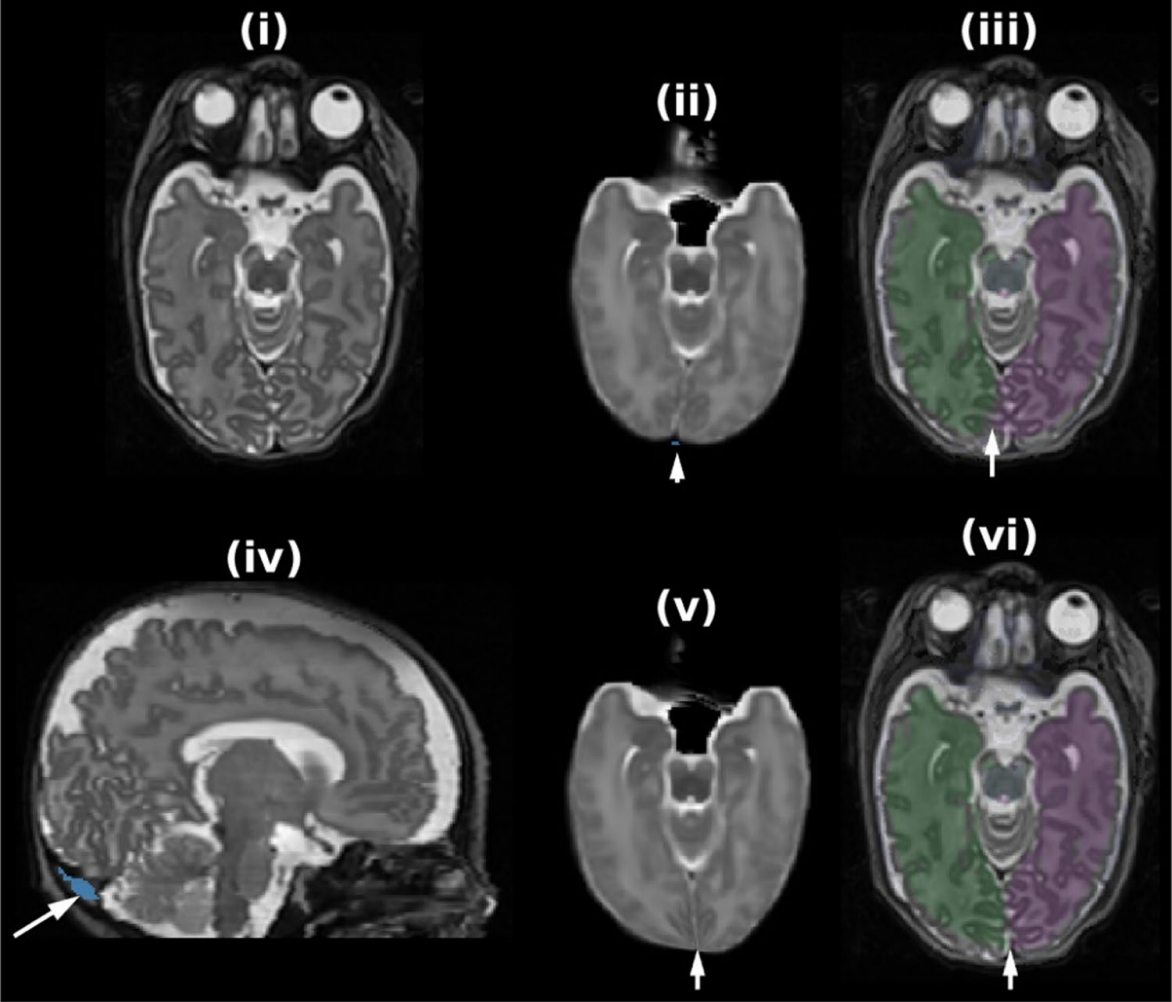
Fissure alignment procedure whose initial input is the subject image (i) and the template image after affine registration (ii). In (iii) inspection of the left/right (purple/green) hemisphere labels reveal misalignment at the posterior fissure with the template left hemisphere penetrating the right hemisphere of the subject image (see the arrowhead). The blue regions shown in (arrowhead ii) and (arrowhead iv) show the manual/automatically delineated “posterior fissure” masks, respectively. Figures (v) and (vi) depict the result of the non-linear fissure mask + intensity warping which demonstrates the correct alignment of the template image hemispheres post-warping (arrowhead vi)

The 40-week *ALBERTs T*_*2*_-weighted template image is linearly and non-linearly registered to the native subject space. The accuracy of linear registration is dependent on the initial transforms chosen and the method used. To increase the robustness of results, the pipeline uses multiple registration methods, giving multiple candidate registrations, of which the best is selected. Specifically, the methods executed are as follows: 1) *ANTs* with center-of-gravity translation initialization, 2) *ANTs* with multi-start affine (*antsAI*) initialization, and 3) FLIRT (Jenkinson et al., 2002). The *ANTs* registration parameters are defaults from the antsRegistrationSyN.sh script within *ANTs*. The *FLIRT* tool performs affine registration in two stages as follows: 1) mutual information-based registration followed by 2) correlation ratio-based registration weighted by template and native brain masks. The registration that produces the superior mutual information cost is chosen. The non-linear registration is performed with *ANTs* and is composed of two stages: 1) a coarse transformation that primarily aims to correct misalignment of the posterior interhemispheric fissures after affine registration (see Fig. 5(i-iii) for an example), while also, to a lesser extent, using image intensities to drive registration globally, and then 2) a fine-scale alignment based on image intensities.

The first stage uses a *T*_*2*_-weighted intensity channel, for global registration, and a “fissure mask”, which are the regions within the indentation formed by the interhemispheric fissure at the occipital lobe (see Fig. 5(ii) and (iv)), to align the hemisphere boundaries. The “fissure mask” is initialized as the voxels inside the convex hull of the brain mask, but not in the brain mask. The template image was initialized in this manner and then manually edited to remove voxels not in the occipital region. The “fissure mask” for a subject image is created using the same initialization as the template image and then masked using the dilated, affine-transformed template “fissure mask” to remove non-occipital voxels; Fig. 5(iv) shows an example for a subject image. The registration parameters included heavy image smoothing to suppress fine details and to encourage overlap of the masks. The *SyN* parameters were chosen to make the deformation contain fluid and elastic components to mostly maintain the original geometry of the template brain (see Table 2 for details). Figure 5(v) and (vi) shows the final registration where the template’s left hemisphere (purple label) no longer encroaches into the subject’s right hemisphere and the fissure (arrowhead) is at the correct position.

**Table 2:** Parameters for ANTs’ non-linear transformation of ALBERTs template to subject space

Stage	Transform	Update field smoothing	Total field smoothing	Iterations	Shrink factors	Smoothing	Cost functions, channels, weighting
1	SyN	3	1	100	2	4 voxels	MI, *T*_*2*_, 0.3
							Demons, Posterior Fissure mask, 1
2	SyN	1.5	0	100 × 30 × 30	4 × 2 × 1	2 × 1 × 0 voxels	MI, *T*_*2*_, 1
							LCC, *T*_*2*_, 1
							MI, Hemisphere, 0.5

The second stage of the registration uses a *SyN* transformation with fluid components. The registration is mostly driven by equally weighted MI + LCC cost functions on the *T*_*2*_-weighted intensities. To inhibit contralateral deformation at the midline, a “hemisphere” image was constructed on the ALBERTs with values 1 or 2 depending on a voxel being left or right of the midline. This image along with its affine transformed version forms an image pair with a MI cost function. See Table 2 for details on all the parameters chosen.

The resultant affine and non-linear transforms are applied to project the template *ALBERTs* and M-CRIB-S data into subject space.

We estimate the *ALBERTs*’ 9-label “tissue type” segmentations (Gousias et al., 2012) in the subject space using *DrawEM* (Makropoulos et al., 2014) applied to the subject image and transformed probability maps (see Fig. 2 for a depiction of a labelled subject indicated by the box “DrawEM tissue labeling”). This labeling is used by various downstream processing steps.

### Registration of M-CRIB-S Training Data for Label Fusion

Each M-CRIB-S training image and its labels are propagated to subject space for label fusion. This registration computes a linear (*Rigid* + *Affine*) and non-linear warp using ANTs. The linear stage is initialized using the composed affine transform as follows: training image → M-CRIB-S template → ALBERTs template → subject image, and then carried out using the same parameters used previously (see Table 2). The non-linear warp is computed using the 3-stage method whose first stage is the same fissure alignment routine described earlier and whose 2nd and 3rd stages use intensity images and mask pairs for the cortical GM, corpus callosum (CC), SP, and lateral ventricle structures. The method for approximate mask generation will now be described.

The CC is typically labelled accurately by the M-CRIB-S majority vote image. Therefore, that label, transformed to the subject space, is used as the CC region for registration.

**Fig. 6:**
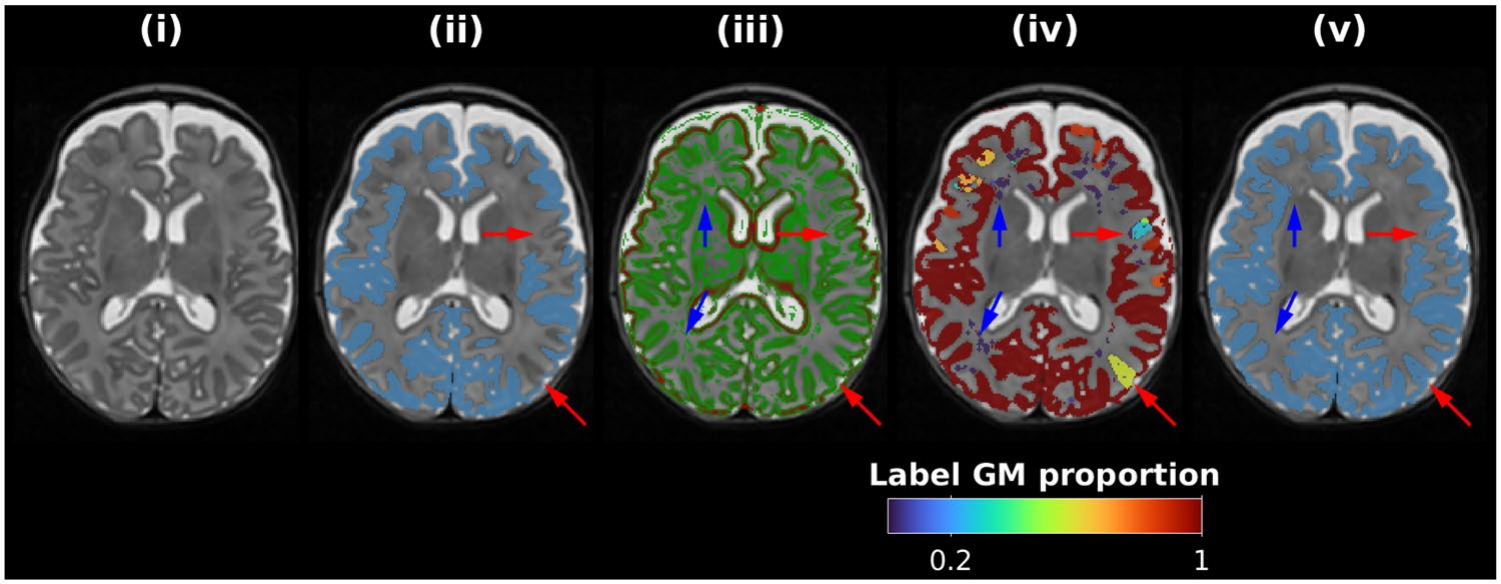
Depiction of approximate GM label mask creation routine. (i) T_2_-weighted image, (ii) DrawEM GM label overlaid (blue) with red arrows highlighting errors, (iii) sign of 2nd derivative of T_2_-weighted image colored as follows: negative (red, locally bright), positive (green, locally dark) or almost zero (transparent, locally uniform). (iv) Connected components of positive 2nd derivative voxels from panel (iii) colored according to the proportion of their perimeter neighboring the original GM label in panel (ii); Red and blue arrows highlight above- and below-threshold connected components, respectively. The final GM label is shown in (v), which shows the result of connected components thresholding

Figure 6 depicts the method for approximate GM mask creation. Firstly, the mask is initialized per the GM region from 9-label “DrawEM tissue label” segmentation (see Fig. 6(ii)). Residual bias or biological intensity variation results in missing sections (Fig. 6(ii)). The locally dark stripe of the cortical GM is highlighted by computing a mask of the positive values of the 2nd derivative of a Gaussian sampled along the image gradient direction (green in Fig. 6(iii)). A method is required to reclassify regions of WM that are dark, such as periventricular WM or small hypointense regions, which are initially classified as GM, (blue arrows in Fig. 6(iii)) while retaining cortical GM (red arrows in Fig. 6(iii)). After removing image edge voxels via a Canny edge detector, connected components are computed (see Fig. 6(iv)). For each connected component, its mask is replaced with the proportion of its perimeter neighboring the original, slightly dilated, GM label (see in Fig. 6(iv)). Finally, binary thresholding (0.2 used by default) is applied to this image to generate the final mask (see Fig. 6(v)); note that the dark WM (blue arrows) were removed, and the cortical GM (red arrows) were retained.

**Fig. 7:**
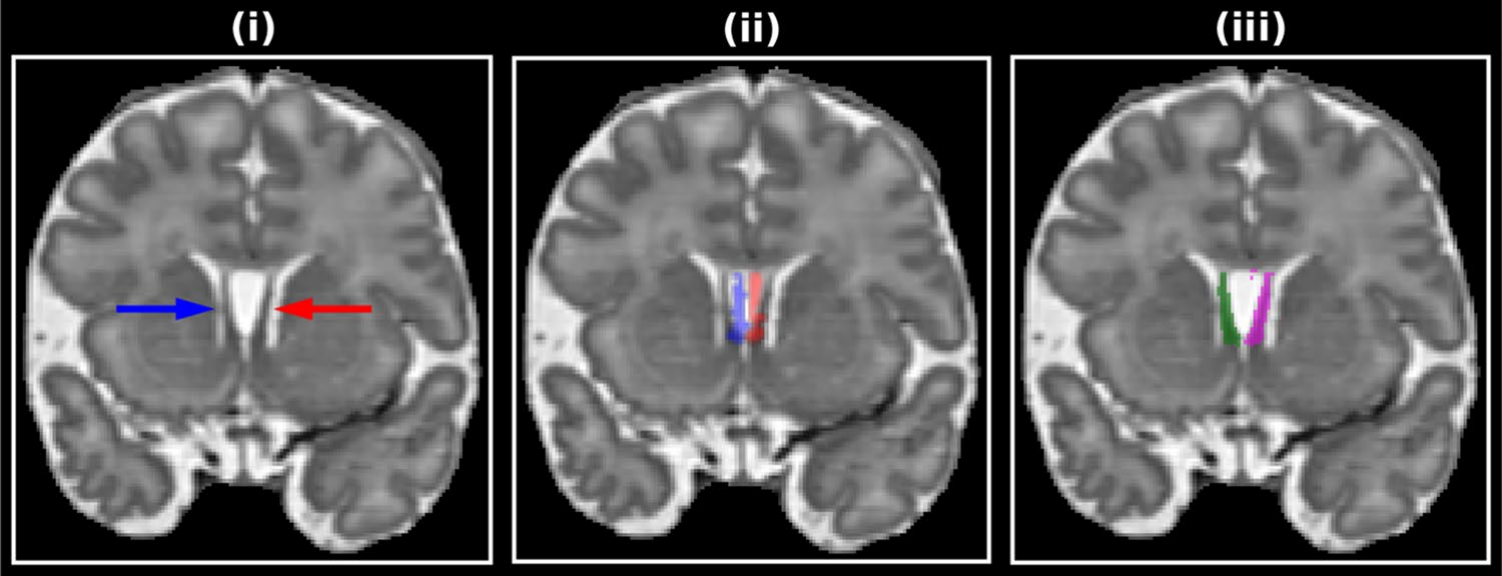
Septum pellucidum (SP) mask generation example. (i) shows the original T2-weighted subject image with the left/right SP denoted by red/blue arrowheads. (ii) shows the template left/right SP masks after registration. (iii) shows the estimated SP masks after lateral search from the masks in (ii)

The SP masks are used to correct misregistration caused by larger cavum septum pellucidi that are not adequately aligned using intensity-based alignment alone. The mask generation technique is depicted in Fig. 7 and will now be described. Left/right SP masks are initialized from transformed masks manually defined on the 40-week ALBERTs template (Fig. 7(ii)). The subject SP are typically offset laterally from those in the template. Therefore, for each voxel in the initial mask, the first relatively dark voxel, determined as either having a positive Laplacian of Gaussian value or being relatively dark compared to their neighbors, are added to the subject masks (see Fig. 7(iii)). Note this is not the final SP segmentation, it is rather a guide for registration, so small errors are acceptable.

The approximate lateral ventricles mask generation method will now be described. The initial mask is the *DrawEM* “ventricles” label. Connected components that do not coincide with the M-CRIB-S majority vote lateral ventricle labels are removed. Voxels that are relatively dark compared to their neighbors, which are not part of the ventricles, are removed using negative values after Laplacian of Gaussian filtering of the *T*_*2*_-weighted image and the dark periventricular ring label described earlier.

The non-linear portion of the training → subject registration is the 4-stage *ANTs SyN*-based registration with parameters shown in Table 3. The first three stages focus on aligning the masked structures, which may require significant deformation, with limited contribution from image intensities. Each stage features a single mask channel with dominant weighting with the following schedule:

1. Lateral ventricles
2. Cortical GM
3. Left and right septum and CC

**Table 3:** Parameters for non-linear transformation of M-CRIB-S training data to subject space

Stage	Transform	Update field smoothing	Iterations	Shrink factors	Smoothing	Cost functions, channels, weighting
1	SyN	1.5	100 × 40 × 10	4 × 2 × 1	2 × 1 × 0 voxels	MI, *T*_*2*_, 0.01
						Demons, Lateral ventricles, 1
2	SyN	1.5	100 × 40 × 10	4 × 2 × 1	2 × 1 × 0 voxels	MI, *T*_*2*_, 0.01
						Demons, Lateral ventricles, 0.01
						Demons, Cortical GM, 1
3	SyN	1.5	100 × 40 × 10	4 × 2 × 1	2 × 1 × 0 voxels	MI, *T*_*2*_, 0.01
						Demons, Lateral ventricles, 1
						Demons, Cortical GM, 0.1
						Demons, LH septum, 1
						Demons, RH septum, 1
						Demons, CC, 1
4	SyN	1.5	70 × 20	2 × 1	0 × 0 voxels	MI, *T*_*2*_, 2
						Demons, Lateral ventricles, 0.2
						Demons, Cortical GM, 0.5
						Demons, LH septum, 0.5
						Demons, RH septum, 0.5
						Demons, CC, 0.5

The fourth stage aligns on all masked structures but gives dominant weighing to image intensities to register non-masked structures.

Given the native *T*_*2*_-weighted image and each registered *T*_*2*_-weighted/label image pair from the M-CRIB-S training data, the tool antsJointFusion (Wang & Yushkevich, 2013) is executed to assign maximally probable labels to each brain voxel. Briefly, the method applies weighted voting based on the goodness-of-fit of the local image patch in the subject image to local image patches of the training images within a search radius; the patch size and search radius are user-defined parameters. Parameter values chosen here are as follows: patch size = 2 mm, search radius = 2 mm near the GM (using morphological dilation on the GM labels) and 1 mm elsewhere, goodness-of-fit = Pearson’s Correlation, defaults otherwise. 

The final output label image is named: TissueSegMCRIBS/ < foo > / < foo > _labelfusion_dkt.nii.gz, and is copied immediately to TissueSegMCRIBS/ < foo > _labelfusion_dkt_edited.nii.gz. The latter is used as input for future processing and is the file to be edited for error correction.

#### Cortical Surface Extraction

Cortical surface extraction is performed using the Deformable-based (Schuh et al., 2017) pipeline described previously (Adamson et al., 2020). The methods and major updates to the original version (Adamson et al., 2020) are described below. The processing directive for this step is --surfrecon.

### Postprocessing Label Fusion Outputs

The Deformable-based (Schuh et al.) cortical surface extraction technique used in M-CRIB-S requires a label image with the following regions: 1) Brainstem + cerebellum, 2) cortical GM, 3) left/right cortical WM, 5) left/right lateral ventricles; named SurfReconDeformable/ < foo > /recon/regions.nii.gz. Note that the accuracy of the GM label is unimportant, since the inner surface is generated from the white matter label and the pial surface is primarily generated from image intensity information. The brainstem + cerebellum, cortical GM and lateral ventricle labels are directly translated from the label fusion output.

The left/right cortical WM labels are modified so that they artificially meet at the midline and, thus, their border approximates the medial wall; this is required to create the spherical topology of the cortical surfaces. Voxel labels are reassigned using the label fusion output to the WM tissue type as follows:

- Voxels added after morphological closing (25 iterations) of the CC and Thalamus labels
- Lateral ventricles, thalamus, caudate, putamen, hippocampus, choroid plexus, amygdala, accumbens, ventral diencephalon (ventralDC) labels
- Dilated (2 iterations) 3rd ventricle label

Finally, each voxel of this binary mask is assigned to the left or right hemisphere. This is accomplished by firstly warping a majority vote “ribbon” image, i.e., a label image with left/right WM labels artificially meeting at the midline, from template to subject space. Each voxel is assigned to the warped majority vote hemisphere label that has a lower Chessboard distance.

Segmentation errors in the label fusion output often appear near the cerebral cortex, since highly variable folding features will often align incorrectly based on image-based registration. Additionally, intensity patterns, the goodness-of-fit metric for label fusion, for folding features are difficult to identify correctly. For example, the intensity pattern bright-dark-bright-dark-bright may represent a sulcus (WM-GM-CSF-GM-WM) or gyrus (CSF-GM-WM-GM-CSF). Automatic methods for addressing commonly seen errors have been included as post-processing. These will now be described.

**Fig. 8:**
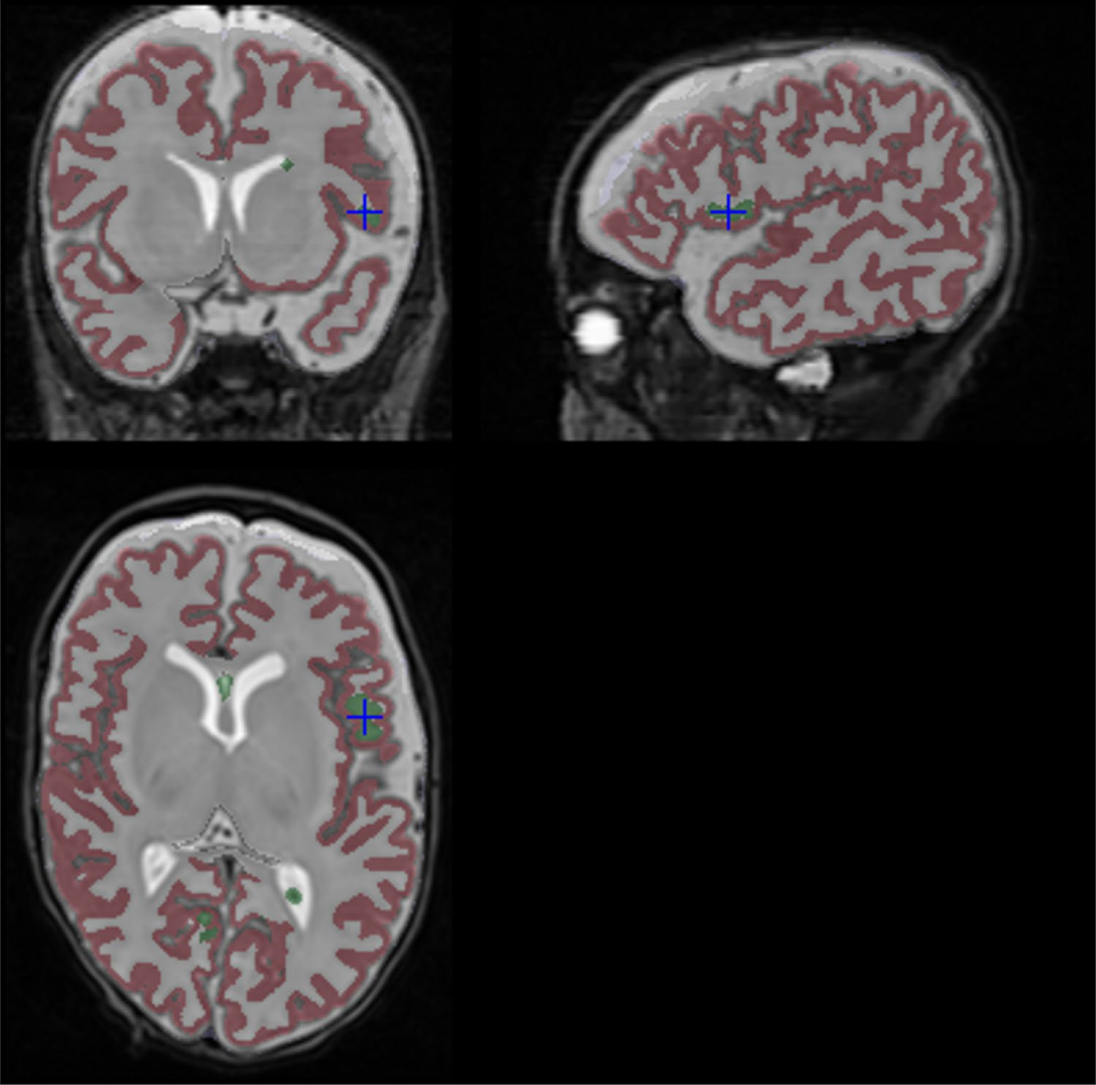
Example of a connected component labelled by the label fusion as CSF (green and blue crosshair), relabelled as WM by being connected to the largest WM connected component

WM voxels are erroneously labeled as CSF near the brain boundary. These errors occur mostly in locations where cortical folding landmarks are misaligned after image registration. To correct these errors, we identify connected components of CSF voxels that touch the main WM label component are reassigned to the corresponding hemisphere WM label. Figure 8 shows an example of this fix applied.

**Fig. 9:**
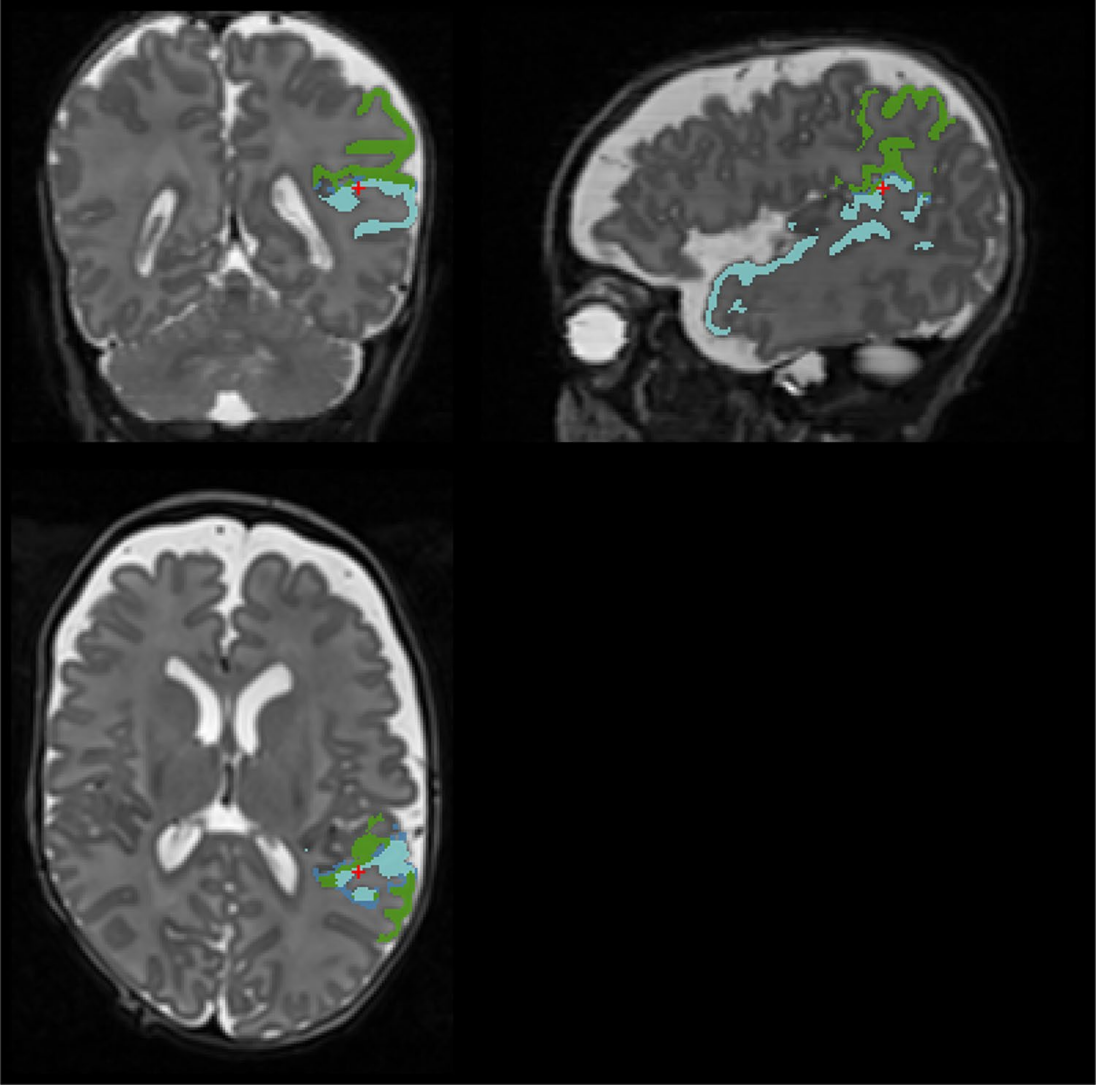
Label pair boundary voxel label replacement example for Superior Temporal (blue) and Supramarginal (green) label. The label of the voxels between the space where the labels meet (the red crosshair) will be replaced with the CSF label

CSF voxels between sulcal banks will be mislabeled as WM. In cases where these errors occur in sulci with unlike labels on each bank, the WM voxels that overlap after dilation of the two labels are reset to CSF. For example, Fig. 9 shows the Superior Temporal and Supramarginal regions where the red crosshair is the approximate location of where voxel labels would be changed; Figure S1 shows all pairs of labels for which this fix is applied.

Finally, added voxels are assigned to their hemisphere upon computing Euclidean distance transforms for each of the transformed ribbon labels. Labels are then translated into those mentioned previously: 1) Brainstem + cerebellum, 2) cortical GM, 3) left/right cortical WM, 5) left/right lateral ventricles; and saved to the image SurfReconDeformable/ < foo > /recon/regions.nii.gz. 

### Surface Extraction

**Fig. 10:**
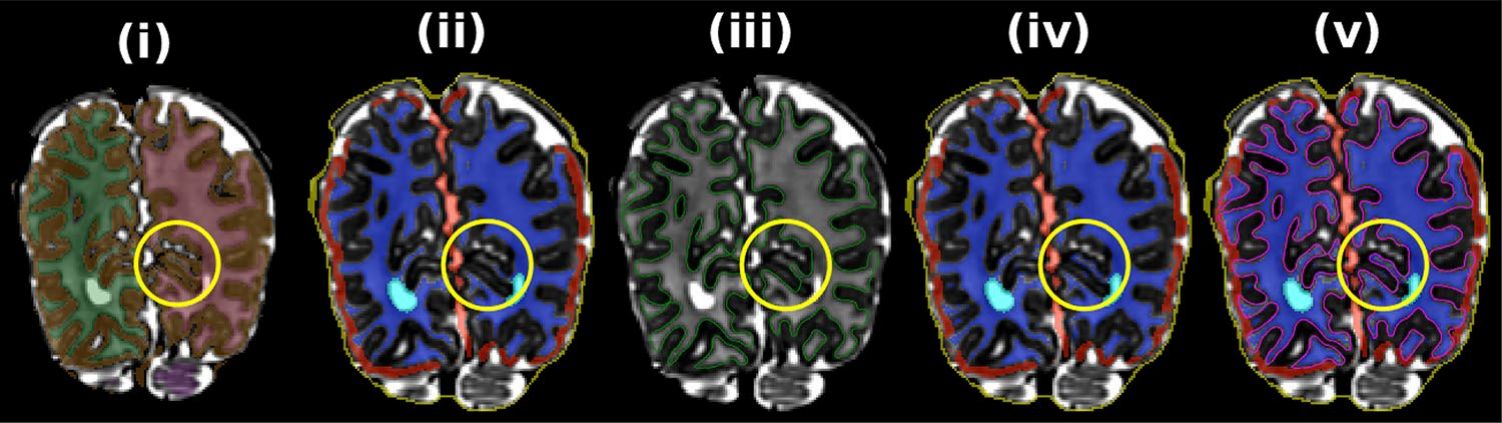
WM surface construction steps. (i) With “regions.nii.gz” overlaid. The yellow circle highlights a region of interest where the proposed fix had a noticeable effect

The left/right hemisphere WM surfaces are initialized using the boundaries respective voxel-based cerebral WM labels followed by iterative refinement towards falling *T*_*2*_-weighted intensity edges which indicate WM to GM transition. The pial surfaces are initialized using the WM surfaces and deformed towards rising *T*_*2*_-weighted image intensity edges which indicate GM to CSF transition; this is unchanged from the previous version. An error that occurred in the previous version is that partial volume contamination in thin gyri would often cause WM voxels to be mislabeled as GM. WM surfaces would thus not penetrate appropriately. The updated version adds two “force” images whose values modulate inward (positive, yellow/red) or outward (negative, blue) movement in the surface normal direction to fix these errors (see Fig. 10). The first force image encourages outward movement from the WM label and inward movement away from the CSF and brain boundary (Fig. 10(ii)). A second WM surface deformation step is performed with an additional image-based outward normal force for thin strands of relatively bright voxels underneath the first WM surface (Fig. 10(iv)). This force image is restricted to the pericalcarine cortex label of the cortex since this area commonly has penetration issues and globally adding this force often causes errors. After a second deformation, improved penetration of the WM surface can be observed (Fig. 10(v)).

The pial surfaces are constructed in the same fashion as the previous version.

After white and pial surfaces are constructed, the processing directive --autoreconaftersurf performs surface inflation, spherical projection, surface-based registration, cortical parcellation, computes measures of cortical geometry and refines the original voxel segmentations using the surfaces.

### Error Reporting and Manual Editing

The surface reconstruction pipeline will sometimes produce erroneous outputs, such as surfaces with topological violations. These error states are typically caused by uncorrected segmentation errors. The pipeline facilitates a manual editing protocol to correct problems, to create correct outputs; fully described in the software documentation doc/manual_editing/Readme.md. Firstly, the user executes the script surfrecon_last_file_made.sh which produces a tab-delimited table of subject IDs and the last file created in the temporary directories. If the file is named ‘pial-6.vtp’ then the pipeline has completed successfully, while other file names indicate an error occurred and manual editing is required. A collection of scripts is available to visualize errors according to the last-created file using the Freesurfer tool freeview. For example, if “pial-5.vtp” is the last file created, there is a script called DisplayPial5Error. The scripts load the bias corrected T2-weighted volume and the label input file SurfReconDeformable/ < foo > /recon/regions.nii.gz described previously.

**Fig. 11:**
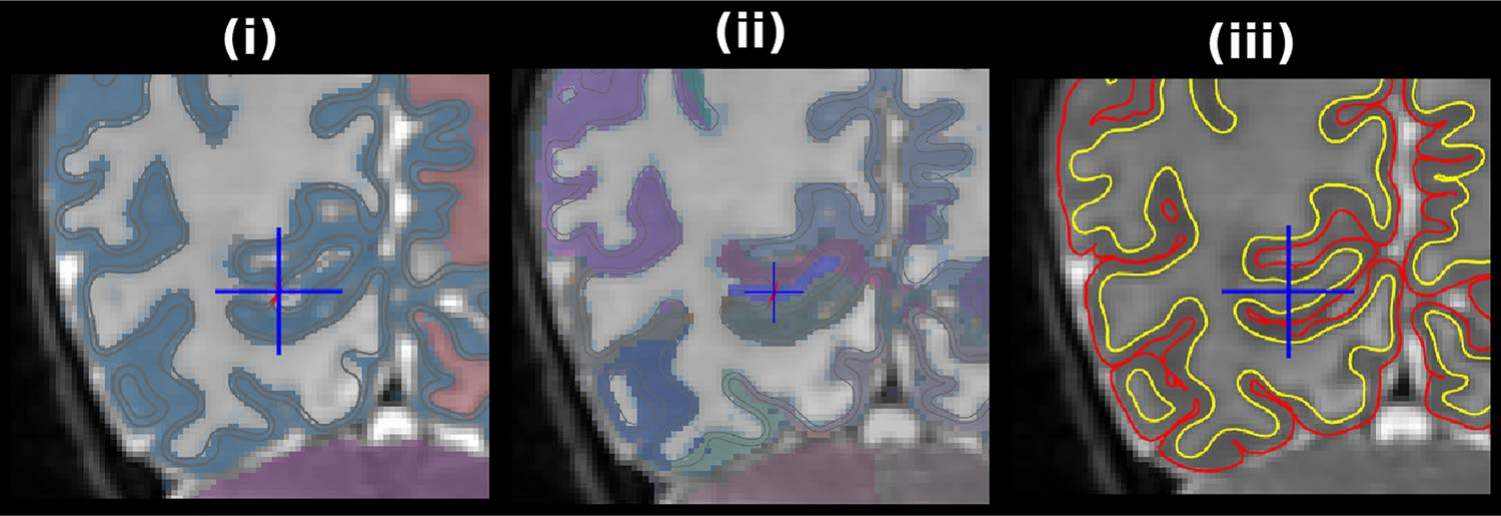
Example of manual edits to fix a “pial-5” error; the error is an erroneous occlusion of the white matter (WM) surface (crosshair in (i)). In (i) the “inner” band is the final white matter surface while the outer band is the initial configuration of the pial surface after outward projection. The red edges underneath the cross hair denote vertices that are members of self-intersecting faces. The voxels are labelled as RH WM (white), LH WM (red), GM (blue), cerebellum (purple). (ii) The edited image labelfusion_dkt_edited.nii.gz with edited RH WM voxels shown as blue underneath the crosshair. (iii) Final white (yellow) and pial (red) surfaces after rerunning the surface extraction pipeline

Figure 11 shows an example of a “pial-5” error, where the last file created is pial-5.vtp. Figure 11(i) is centered on the location of a topology error in the pial surface shown in red. Here, the WM labels in the gyrus should have been contiguous but were segmented by GM labelled voxels. The red faces in the pial surface are self-intersecting and hence indicate a topology violation. Figure 11(ii) shows the TissueSegMCRIBS/ < foo > / < foo > _labelfusion_dkt_edited.nii.gz, overlaid with a manual fix that was applied to fill in the WM labels in the gyrus. To ensure these edited values are not replaced by automated preprocessing prior to future surface extraction, the sentinel values of 254 and 255 should be used in place of the original values of 2 and 41 for LH WM and RH WM, respectively. Figure 11(iii) shows that the surfaces correctly penetrate the gyrus after rerunning surface extraction post-fix.

### Evaluation

**Fig. 12:**
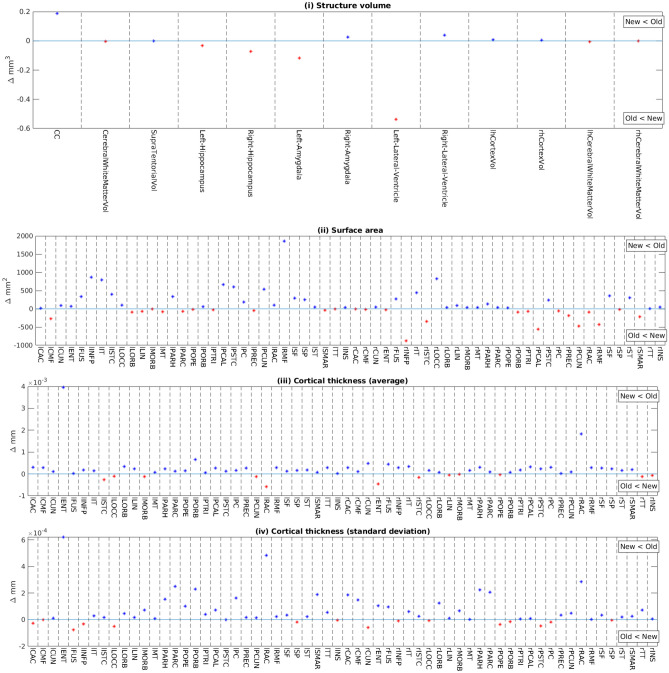
Difference in variances between methods (old – new) for voxel-based structure volumes (i) and geometric properties of DKT cortical regions (ii-iv) that are as follows: surface area (ii), mean cortical thickness (iii), standard deviation of cortical thickness (iv)

To characterize reliability and performance of the revised software, the software was executed on the dHCP release 3 (Hughes et al., 2017) dataset, containing 885 images. Firstly, we conducted a pseudo-repeatability analysis to investigate reliability improvements. In the absence of gold-standard labels and surfaces, we quantified reliability to be the variance of age-related changes of structure volumes and parcel-wise cortical surface area and curvature. Images from a total of 27 (18 male) twice-scanned subjects were chosen for comparison. Birth age: (M 33.8, SD 3.5266 weeks PMA), mean scan ages (36, SD 1.695 weeks PMA at first scan, 41, SD 1.4213 weeks PMA at second scan). Criteria for selection were 1) a minimum first-scan age of 34 weeks, and 2) both timepoints ran successfully on both software versions without manual intervention. Figure 12 shows the differences of variances of fitted slopes of age to (i) compatible structure volumes, and cortical surface properties (ii-iv). For the structure volumes (panel (i)), variances are preserved for large structures (total and hemispheric white and gray matter volumes), improved for the CC and variable for the lateral ventricles when comparing the new with the old method. For cortical surface parameters (panels (ii-iv)), most regions exhibit lower slope variances for the new method. Secondly, the success rate of surface reconstruction without topological errors was 98% (845/885).

Execution times of the proposed pipeline were evaluated for 10 randomly selected dHCP subjects on an Intel Xeon Gold 6240 machine (see Table S1). Mean total execution time was 8246 s (SD 1312 s). The most computationally expensive component of the pipeline was “Individual training to native registration” (mean 3609 s, SD 563 s).

**Fig. 13:**
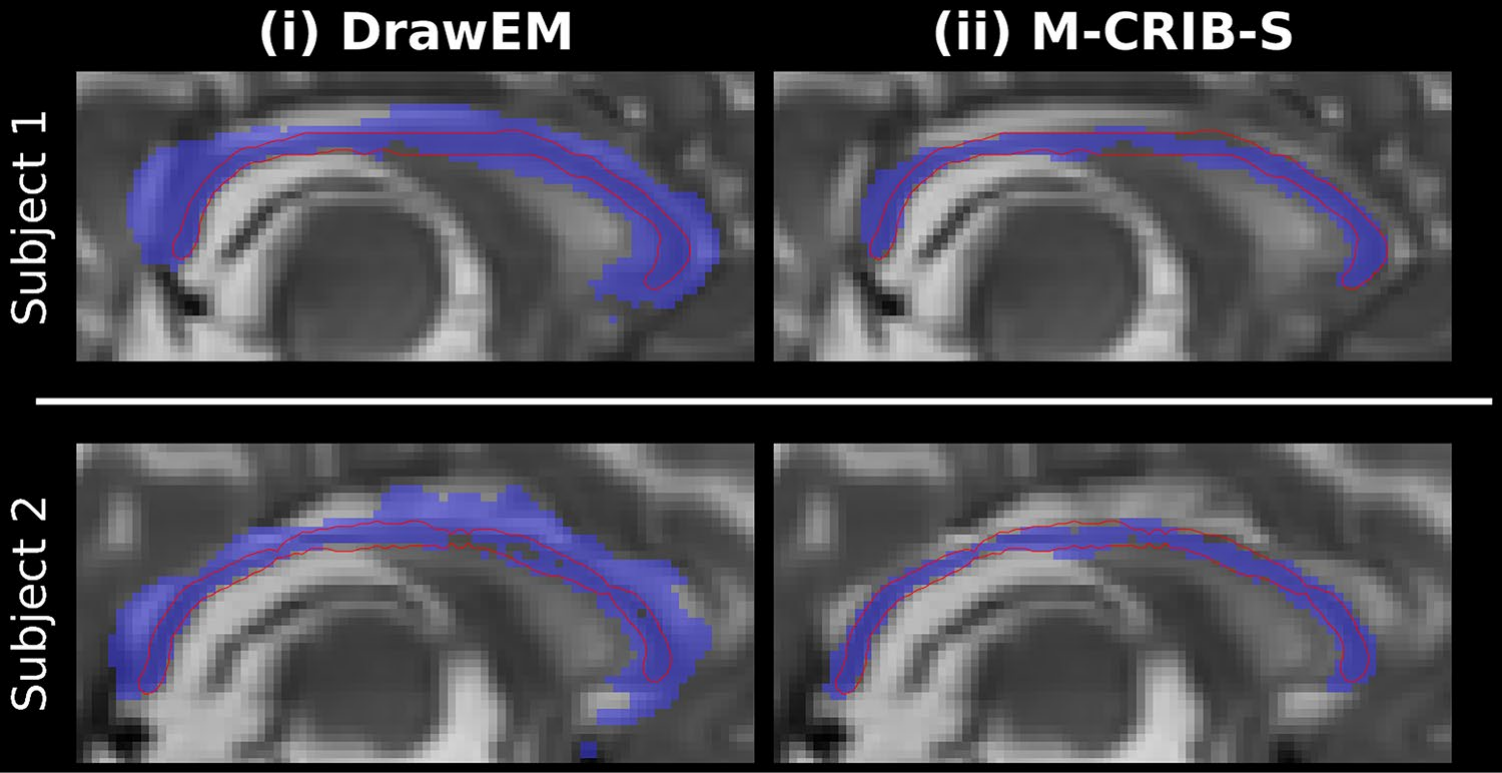
Corpus Callosum segmentation improvements from the original software version using DrawEM (i) to the new software version using M-CRIB-S (ii) for two example subjects. The estimated CC labels are shown in purple with the ground truth, manually delineated by author C.A., shown by outline as red contours

For the CC, the old software version would, in most cases, produce erroneous and variably overestimated segmentations; the new software version labels do not have this issue (see Fig. 13 for a qualitative example of two subjects from the dHCP collection).

## Discussion

The updated M-CRIB-S package was shown to deliver improved CC segmentation and less variable cortical surface parameters, which is also thought to equate with improved cortical surface extraction and reliable parcellation.

Regarding the CC results, the previous version of the software used *DrawEM* version 1.1. A later version of the *DrawEM* software (version 1.3), released after the old version of the software was developed, contains an “improvement for CC segmentation” that may address the issue shown in Fig. 13.

There are some limitations that will possibly be addressed with future updates. Subjects that have lateral ventricles enlarged to the point of being significantly different to those in the training data may not be processed successfully. A pipeline with altered registration parameters to attempt to address large ventricles is planned. There are some labels that were included to improve segmentation outputs but are not considered reliable for volumetric measures, including choroid plexus, CSF, and “skull”. This updated M-CRIB-S package was evaluated only using high-quality datasets, and researchers would need to evaluate it on their own datasets.

The protocol for error handling involves 1) terminating processing immediately with a diagnostic message, followed by 2) manual editing of label fusion outputs to correct problems, and 3) rerunning the surface extraction step. Empirically, surface topology errors and incorrect placement are mostly collocated; thus, topology checking acts as an error detection mechanism. By providing scripts to highlight errors for correction in the program *Freeview*, the final output should be a faithful reconstruction of cortical geometry.

The *DrawEM* pipeline recently included the M-CRIB training data as an option for whole-brain labelling. The training data, however, was not downloadable so it was not possible to evaluate this feature in *DrawEM*.

## Conclusion

We have presented an update to the *M-CRIB-S* software package that achieves two goals: 1) A M-CRIB-based whole-brain segmentation that is compatible with *Freesurfer* and, 2), improved inner surface extraction, enabling more accurate cortical surface measures to be obtained from neonatal brain images.

## Information Sharing Statement

The software, which includes all training data, is available for download from GitHub at (https://github.com/DevelopmentalImagingMCRI/MCRIBS) and as a Docker container at (https://hub.docker.com/u/developmentalimagingmcri/mcribs).

### Supplementary Information

Below is the link to the electronic supplementary material.

Supplementary file1 (DOCX 1603 KB)

Supplementary file2 (DOCX 18 KB)

## DKT Cortical Region Abbreviations

CAC: Caudal Anterior Cingulate
CMF: Caudal Middle Frontal
CUN: Cuneus
ENT: Entorhinal
FUS: Fusiform
INFP: Inferior Parietal
IT: Inferior Temporal
ISTC: Isthmus Cingulate
LOCC: Lateral Occipital
LORB: Lateral Orbitofrontal
LIN: Lingual
MORB: Medial Orbitofrontal
MT: Middle Temporal
PARH: Parahippocampal
PARC: Paracentral
POPE: Pars opercularis
PORB: Pars orbitalis
PTRI: Pars triangularis
PCAL: Pericalcarine
PSTC: Postcentral
PC: Posterior Cingulate
PREC: Precentral
PCUN: Precuneus
RAC: Rostral Anterior Cingulate
RMF: Rostral Middle Frontal
SF: Superior Frontal
SP: Superior Parietal
ST: Superior Temporal
SMAR: Supramarginal
TT: Transverse Temporal
INS: Insula
