# Supplementary material for: Updates to the Melbourne Children’s Regional Infant Brain Software Package (M-CRIB-S)
Source: Neuroinformatics. 2024 Mar 16;22(2):207–23. doi: 10.1007/s12021-024-09656-8 (PMC11021251; doi:10.1007/s12021-024-09656-8)
Supplement: Supplementary file 1 — Supplementary file1 (DOCX 1603 KB) [file 12021_2024_9656_MOESM1_ESM.docx]

Insula and Precentral

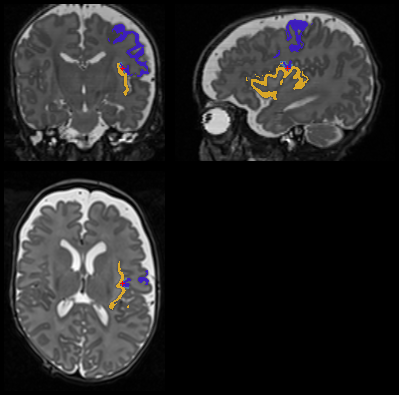

Insula and Postcentral

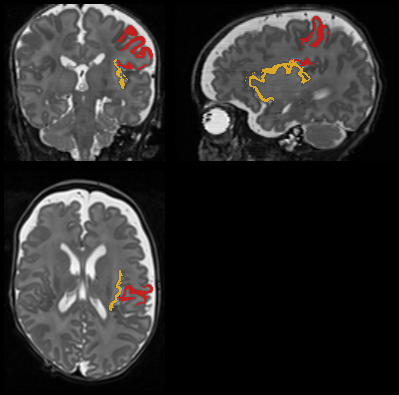


Insula and Transverse Temporal

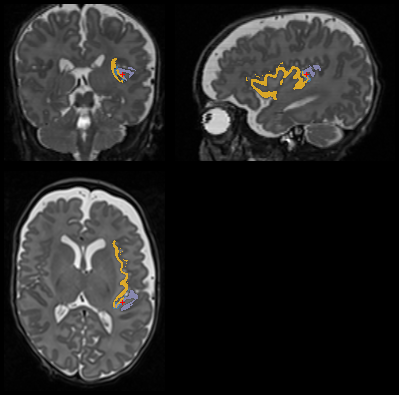

Postcentral and Transverse Temporal

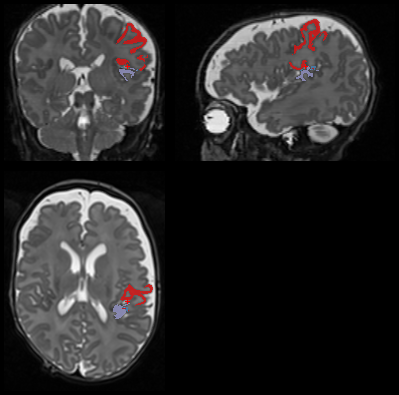


Postcentral and Superior Temporal

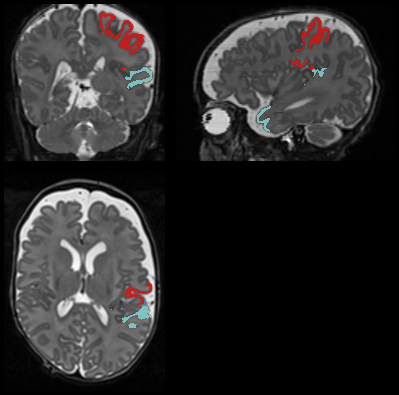

Isthmus Cingulate and Lingual

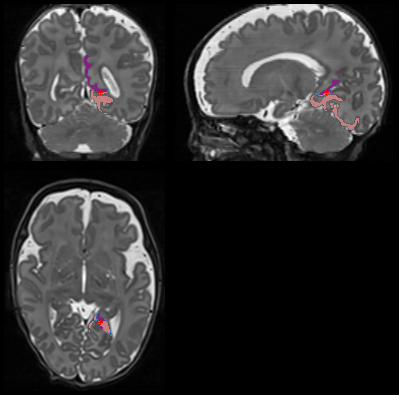


Middle Temporal and Superior Temporal

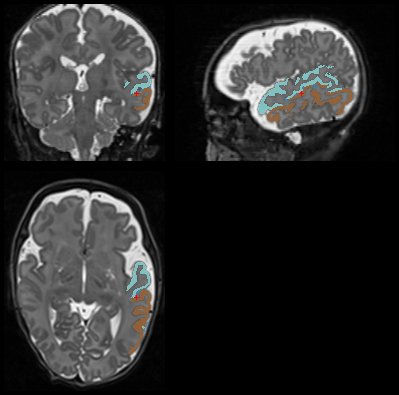

Insula and Superior Temporal

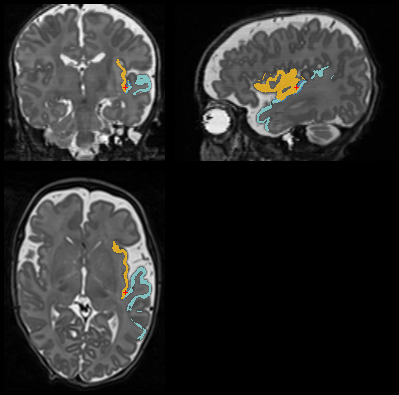


Cuneus and Pericalcarine

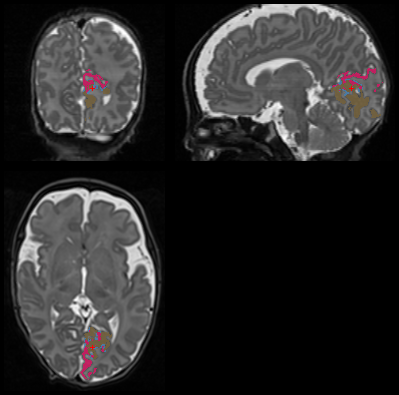

Cuneus and Precuneus

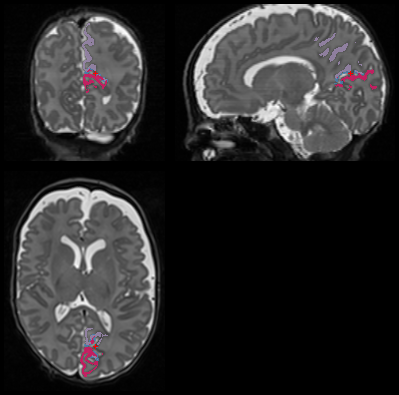


Postcentral and Precentral

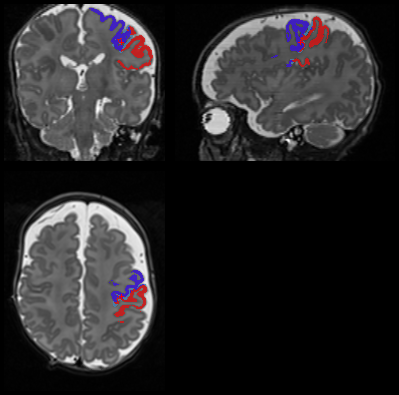

Cuneus and Superior Parietal

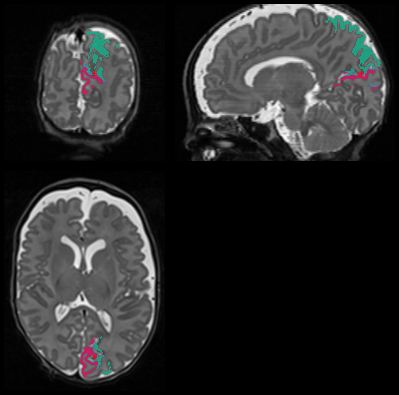


Inferior Parietal and Superior Parietal

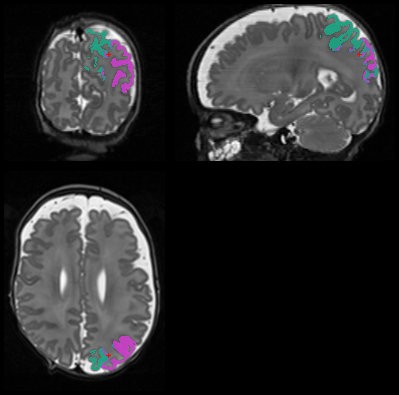

Pars opercularis and Insula

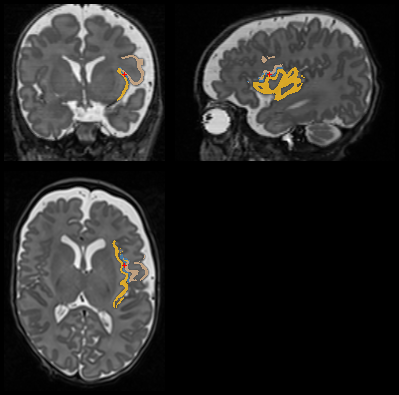


Pars opercularis and Precentral

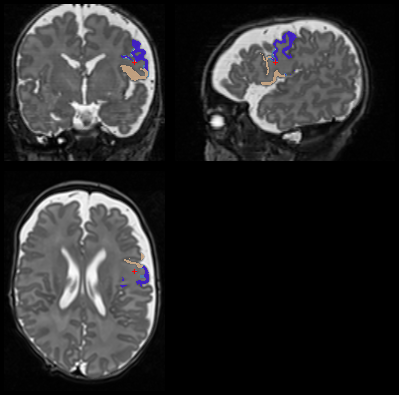

Pars triangularis and Rostral Middle Frontal

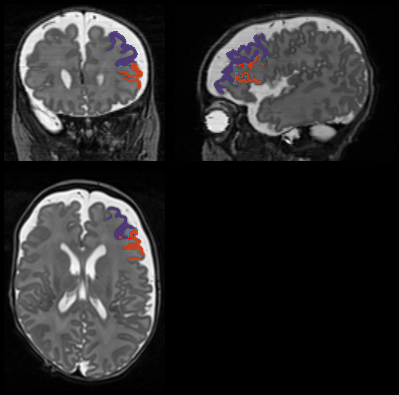


Superior Temporal and Supramarginal

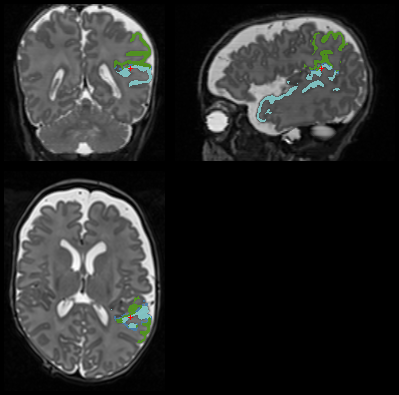


Figure S1: Pairs of labels used for CSF fixing, left hemisphere shown. Colours are from the Freesurfer colour scheme. Light blue voxels in between the labels denote those set to CSF by the method.
