# Supplementary material for: Updates to the Melbourne Children’s Regional Infant Brain Software Package (M-CRIB-S)
Source: Neuroinformatics. 2024 Mar 16;22(2):207–23. doi: 10.1007/s12021-024-09656-8 (PMC11021251; doi:10.1007/s12021-024-09656-8)
Supplement: Supplementary file 2 — Supplementary file2 (DOCX 18 KB) [file 12021_2024_9656_MOESM2_ESM.docx]

# Execution time evaluation for dHCP subjects

Test machine: Intel Xeon Gold 6240 (18 cores, 36 threads), 128GB RAM, ubuntu 22.04

Command sequence:

1. MCRIBReconAll --parallel --tissueseg -openmp 36
2. MCRIBReconAll --surfrecon -openmp 36
3. MCRIBReconAll --autoreconaftersurf -openmp 18

| **Subject** | **Template to training registration** | | **DrawEM tissue labeling** | **Individual training to native**  **registration** | **ANTs label fusion** | **Cortical surface reconstruction** | **Freesurfer-like postprocessing** | **Total^†^** |
| --- | --- | --- | --- | --- | --- | --- | --- | --- |
|  | **Best affine** | **Nonlinear** |  |  |  |  |  |  |
| **1** | 124 | 718 | 1313 | 3697 | 163 | 1862 | 496 | 8535 |
| **2** | 133 | 757 | 1429 | 3986 | 172 | 1923 | 542 | 9111 |
| **3** | 131 | 785 | 1571 | 4186 | 180 | 1955 | 486 | 9471 |
| **4** | 133 | 811 | 1696 | 4333 | 185 | 2124 | 522 | 9987 |
| **5** | 114 | 578 | 948 | 2839 | 127 | 1449 | 401 | 6584 |
| **6** | 114 | 634 | 708 | 3162 | 141 | 1625 | 461 | 6986 |
| **7** | 116 | 582 | 1108 | 2909 | 129 | 1355 | 402 | 6730 |
| **8** | 142 | 792 | 1609 | 4210 | 179 | 2153 | 387 | 9651 |
| **9** | 129 | 700 | 1457 | 3618 | 157 | 1741 | 431 | 8390 |
| **10** | 114 | 624 | 852 | 3150 | 142 | 1548 | 449 | 7018 |
| **mean** | **125** | **698** | **1269** | **3609** | **157** | **1773** | **457** | **8246** |
| **sd** | **10** | **88** | **344** | **563** | **21** | **275** | **53** | **1312** |

Table S1: Execution times, in seconds, for significant pipeline steps. Headings correspond to components in Figure 2. ^†^Includes steps not shown in the table.
